# The factors associated with chronic benzodiazepine use in bipolar patients

**DOI:** 10.1192/j.eurpsy.2021.539

**Published:** 2021-08-13

**Authors:** N. Charfi, M. Ben Abdallah, S. Omri, N. Smaoui, R. Feki, J. Ben Thabet, L. Zouari, M. Maalej Bouali, M. Maalej

**Affiliations:** 1 Psychiatry C Department, Hedi chaker University hospital, sfax, Tunisia; 2 Department Of Psychiatry (c), CHU Hedi CHaker hospital Sfax Tunisia, Sfax, Tunisia

**Keywords:** Bipolar Disorders, benzodiazepine, chronic use of BZD

## Abstract

**Introduction:**

Benzodiazepines (BZD) are widely used in patients with bipolar disorder (BD) and their effectiveness is well documented. Therefore, there are major risks associated with BZD use including abuse and dependence. Those risks can be related to the patients’chacteristics, the particularities of BD and the prescribers.

**Objectives:**

To determine the factors associated with chronic use of BZD in patients with BD.

**Methods:**

We conducted a cross-sectional, descriptive and analytical study among a sample of patients with BD (DSM-5) followed in psychiatric outpatient of Hedi Chaker university hospital in Sfax. We used the Benzodiazepine Cognitive Attachment Scale (ECAB) to determine dependent patients

**Results:**

Among the 61 included patients, 50 (82%) had a chronic use of BZD (> 3 months). They had a mean age of 49.3 years (± 14.02 years) and a low socio-economic level in 44%. The type of BD was dominated by type II (66%). Initial episode type was depressive in 78%. The average number of depressive episodes was 2.92±2.3. A rate of 65.5% of patients have already attempted BZD withdrawal. Chronic BZD use was significantly correlated with BZD dependence (p=0.000), low socioeconomic level (p=0.04), depressive type of the initial episode (p=0.011), the depressive recurrence (p=0.000) and the absence of any attempt to discontinue BZD (p=0.011).

**Conclusions:**

Chronic use of BZD in patients with BD is prevalent. In order to minimize this problem in this population, it is important to enhance programs to improve psychiatrist-prescribing behavior and to use cognitive-behavioral therapies in combination with medication to help withdrawal.

